# Integrative Bulk and Single-Nucleus Analyses Nominate COL5A2 as a CAF/ECM-Associated Marker Associated with PDAC Progression

**DOI:** 10.3390/diagnostics16081205

**Published:** 2026-04-17

**Authors:** Kuan-Ting Lu, Tsung-Ming Chang, Chi-Jen Chang, Ju-Fang Liu

**Affiliations:** 1 Department of Neurosurgery, Shin Kong Wu Ho-Su Memorial Hospital, Taipei 111045, Taiwan; m012822@ms.skh.org.tw; 2 School of Dental Technology, College of Oral Medicine, Taipei Medical University, Taipei 11031, Taiwan; a03441@tmu.edu.tw; 3 School of Medicine, Fu Jen Catholic University, New Taipei City 24205, Taiwan; m002008@ms.skh.org.tw; 4 Division of Pediatric Surgery, Shin Kong Wu Ho-Su Memorial Hospital, Taipei 111045, Taiwan; 5 School of Oral Hygiene, College of Oral Medicine, Taipei Medical University, Taipei 11031, Taiwan; 6 Translational Medicine Center, Shin Kong Wu Ho-Su Memorial Hospital, Taipei 111045, Taiwan; 7 Department of Medical Research, China Medical University Hospital, China Medical University, Taichung 40402, Taiwan

**Keywords:** pancreatic ductal adenocarcinoma (PDAC), COL5A2, bioinformatics, cancer-associated fibroblasts (CAFs), single-nucleus RNA sequencing, extracellular matrix (ECM)

## Abstract

**Background/Objectives**: Pancreatic ductal adenocarcinoma (PDAC) is characterized by an extensive desmoplastic microenvironment; however, reproducible stromal-associated biomarkers linked to disease progression remain limited. This study therefore aimed to identify and validate a biologically relevant stromal/extracellular matrix (ECM)-associated candidate biomarker for PDAC. **Methods**: Three GEO bulk transcriptomic PDAC cohorts (GSE15471, GSE16515, and GSE62452) were integrated for differential expression, functional enrichment, protein–protein interaction, and hub-gene analyses. Candidates identified as a promising biomarker were further evaluated using the following: public proteomic and survival resources; head-to-head receiver operating characteristic (ROC) comparisons against COL1A1, COL3A1, and COL5A1; a progression cohort (GSE43288); and single-nucleus RNA sequencing data (GSE202051). **Results**: Among 206 shared differentially expressed genes, COL5A2 was the only consensus hub retained across multiple network-ranking methods. COL5A2 protein expression was found to be elevated in tumor tissue and associated with worse overall and disease-free survival. In ROC analyses, COL5A2 exhibited stable tumor-versus-non-tumor discrimination across GSE15471, GSE16515, and GSE62452 (AUC = 0.932, 0.760, and 0.782, respectively) and significantly outperformed COL3A1 in two cohorts. In GSE43288, COL5A2 expression increased along the normal–pancreatic intraepithelial neoplasia–PDAC axis and remained positively associated with ECM and cancer-associated fibroblast (CAF) signature scores after adjustment for disease group. Reanalysis of GSE202051 restricted to the original 18 untreated PDAC specimens revealed that COL5A2 expression was concentrated in fibroblast-lineage compartments, with CAFs accounting for the largest overall contribution and myCAFs demonstrating the strongest per-specimen expression enrichment. **Conclusions**: COL5A2 is a reproducible stromal/ECM-associated candidate biomarker linked to PDAC progression, with predominant expression in fibroblast/CAF compartments.

## 1. Introduction

Pancreatic cancer remains one of the most lethal malignancies worldwide, in which pancreatic ductal adenocarcinoma (PDAC) constitutes the leading histological subtype and accounts for the vast majority of pancreatic cancer-related deaths [1,2]. Despite advances in systemic therapy and perioperative management, the overall prognosis of PDAC remains poor due predominantly to the fact that many patients are diagnosed at a locally advanced or metastatic stage, which limits eligibility for curative-intent resection and restricts therapeutic options [3,4]. Even when multimodal treatment is feasible, clinical outcomes remain unsatisfactory because of marked intratumoral heterogeneity, early systemic dissemination, and intrinsic or acquired resistance to therapy [1,5]. Accordingly, there is a pressing need to identify reliable biomarkers that can improve disease stratification, better reflect tumor biology, and support the development of more effective therapeutic strategies [3,6].

A defining biological feature of PDAC is its pronounced desmoplastic tumor microenvironment. This microenvironment is characterized by extensive stromal expansion and dynamic extracellular matrix (ECM) remodeling, which often exceeds the epithelial tumor compartment in volume and profoundly influences disease behavior [7,8]. Cancer-associated fibroblasts (CAFs) are a major stromal component of this microenvironment and exhibit substantial functional heterogeneity, contributing to ECM deposition, immune regulation, tissue stiffness, and tumor cell plasticity in ways that promote invasion, progression, and treatment resistance [9,10]. Beyond providing structural support, the ECM-CAF axis regulates tissue biomechanics and growth factor signaling, thereby shaping malignant progression and therapeutic responsiveness [8,11]. These features make stromal and ECM-related molecules attractive biomarker candidates in PDAC; however, currently available biomarkers are still limited in their ability to robustly capture stromal biology and progression-related remodeling across independent datasets, which constrains their translational utility [3,6,12].

Collagens are central ECM components that organize tissue architecture and regulate cell behavior through receptor-mediated signaling and mechanical cues, making collagen remodeling a hallmark of tumor-associated fibrosis [7,8]. Among fibrillar collagens, the dysregulated expression of collagen-related genes, particularly COL5A2, has been associated with stromal activation and unfavorable prognosis in multiple solid tumors, suggesting critical roles in conserved pro-tumorigenic ECM remodeling programs [13,14]. Recent computational and transcriptomic studies have also linked COL5A2 to invasion, epithelial–mesenchymal transition-like processes, and stromal-tumor crosstalk, which supports the potential relevance of COL5A2 as a biomarker candidate [15,16]. Nevertheless, it remains unclear whether COL5A2 in PDAC is reproducibly associated with disease progression across independent cohorts, whether it retains its association with stromal/ECM programs beyond simple group-wise progression patterns, and whether its expression is preferentially localized to fibroblast/CAF-rich compartments at single-cell or single-nucleus resolution [9,12].

To address these questions, we performed an integrative analysis combining three independent bulk transcriptomic PDAC cohorts, public proteomic and survival-association resources, a progression cohort spanning normal-pancreatic intraepithelial neoplasia (PanIN)-PDAC lesions, and single-nucleus RNA sequencing data. This design was intended to identify reproducible stromal/ECM-associated candidate genes across independent datasets and to determine whether their expression could be biologically contextualized within fibroblast-rich tumor microenvironments. Within this framework, COL5A2 was prioritized for further evaluation as it emerged as the only consensus hub across multiple network-ranking methods and exhibited consistent associations with stromal and progression-related programs. We further examined whether COL5A2 demonstrated supportive protein-level and clinical-association patterns in public resources and whether its signal could be attributed predominantly to stromal/fibroblast compartments. Collectively, this study aimed to evaluate COL5A2 as a biologically grounded stromal/ECM-associated candidate biomarker in PDAC.

## 2. Materials and Methods

### 2.1. Data Sources and Cohort Selection

**Figure 1:**
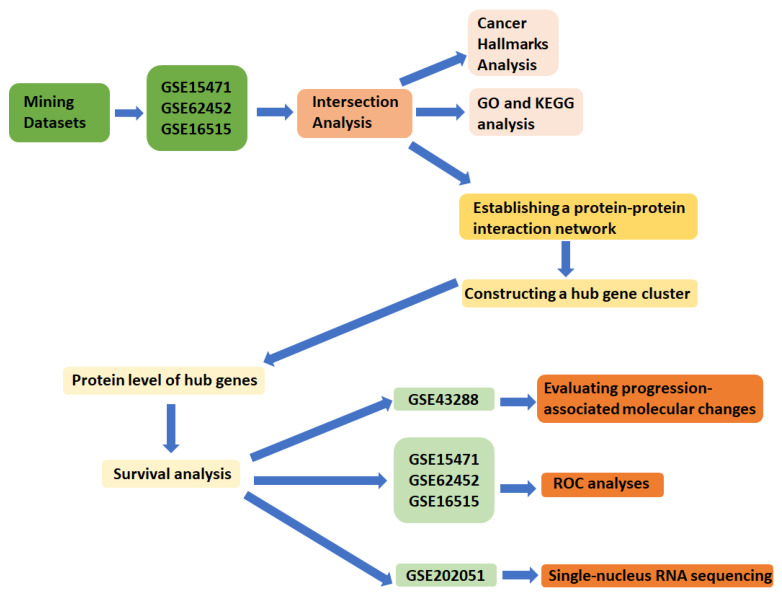
Study workflow for the integrative prioritization and multi-level evaluation of COL5A2 in pancreatic ductal adenocarcinoma (PDAC). Three independent Gene Expression Omnibus (GEO) bulk cohorts (GSE15471, GSE16515, and GSE62452) were used for differential expression analysis and cross-cohort intersection to define robust shared differentially expressed genes (DEGs). These genes were then subjected to Hallmark, Gene Ontology (GO), and Kyoto Encyclopedia of Genes and Genomes (KEGG) enrichment analyses, Search Tool for the Retrieval of Interacting Genes/Proteins (STRING)-based protein–protein interaction (PPI) network construction, Molecular Complex Detection (MCODE) module detection, and cytoHubba hub-gene ranking. COL5A2 was subsequently evaluated using external proteomic and survival-association resources, head-to-head receiver operating characteristic (ROC) analyses against related extracellular matrix (ECM)-associated collagen genes, progression-associated stromal analyses in GSE43288, and cellular-context analyses in the single-nucleus RNA sequencing dataset GSE202051.

Publicly available PDAC transcriptomic datasets were retrieved from the Gene Expression Omnibus (GEO) database (https://www.ncbi.nlm.nih.gov/geo/, accessed on 7 February 2026). Three GEO bulk microarray series with tumor–non-tumor comparisons were included to screen for consistently dysregulated genes: GSE15471 (Affymetrix Human Genome U133 Plus 2.0; 36 tumor and 36 matched non-tumor tissues; including technical replicate arrays), GSE62452 (Affymetrix Human Gene 1.0 ST; 69 tumors and 61 adjacent non-tumor tissues), and GSE16515 (Affymetrix Human Genome U133 Plus 2.0; 36 tumors and 16 non-tumor tissues, including 16 matched pairs). Using multiple public cohorts enabled us to evaluate the reproducibility of differential-expression signals across studies, thereby strengthening the robustness and interpretability of subsequent analyses (Figure 1).

### 2.2. Differential Expression Analysis and Thresholds

Differential expression analysis for GSE15471, GSE16515, and GSE62452 was performed using GEO2R, which implements the Linear Models for Microarray Data (limma) pipeline (R 4.2.2; Biobase 2.58.0; GEOquery 2.66.0; and limma 3.54.0). GEO2R was applied to the GEO-hosted processed expression matrices provided for each dataset. Differentially expressed genes (DEGs) were defined using Benjamini–Hochberg false discovery rate (FDR) correction with thresholds of adjusted *p* < 0.01 and|log2FC| ≥ 1.

### 2.3. Identification of Robust DEGs Across Cohorts

To prioritize consistently dysregulated genes and reduce dataset-specific bias, DEG lists from the three GEO cohorts were intersected using a Venn diagram strategy (InteractiVenn, https://www.interactivenn.net/, accessed on 14 March 2026) [17]. Genes commonly present in all three DEG datasets were defined as robust candidates and retained for downstream hub-gene prioritization and validation analyses.

### 2.4. Cancer Hallmark Enrichment Analysis

To investigate the biological relevance of the 206 robust DEGs shared across the three GEO cohorts, cancer hallmark enrichment was performed using CancerHallmarks.com (https://cancerhallmarks.com/, accessed on 14 March 2026) [18]. The DEG list was uploaded to the platform, and enrichment for predefined hallmark categories was assessed based on multiple-testing–adjusted *p*-values provided by the tool. Hallmarks with significant enrichment after *p*-value correction were interpreted as cancer-related processes potentially represented by the robust DEG dataset. The resulting output was visualized as a Hallmark Enrichment Plot generated by the platform.

### 2.5. Gene Ontology and Pathway Enrichment Analysis

To further investigate functional categories and pathway-level mechanisms, enrichment analyses were conducted using ShinyGO v0.85.1 (https://bioinformatics.sdstate.edu/go/, accessed on 14 March 2026) [19]. The same set of 206 robust DEGs was uploaded for analysis. Functional annotation was assessed using Gene Ontology (GO) terms—including Biological Process, Cellular Component, and Molecular Function—and Kyoto Encyclopedia of Genes and Genomes (KEGG) pathway analysis. Enrichment significance was determined using an FDR cutoff of 0.05, and the minimum pathway size was set to 10 (default setting). ShinyGO outputs were used to summarize biological processes, cellular localization, molecular functions, and pathway-level associations.

### 2.6. STRING-Based PPI Network Analysis

To explore the interaction landscape among the shared robust DEGs, a protein–protein interaction (PPI) network was constructed using the Search Tool for the Retrieval of Interacting Genes/Proteins (STRING) v12.0 database (https://string-db.org/, accessed on 14 March 2026) [20]. The shared DEG list was uploaded, and the network was generated using a stringent confidence threshold (minimum required interaction score = 0.7; high confidence). The resulting network was used for downstream module detection and hub-gene prioritization. The full set of shared DEGs, rather than a subset restricted to genes from selected enriched pathways, was used for PPI analysis to avoid pathway-preselection bias and to preserve the global interaction structure of the robust cross-cohort DEG dataset.

### 2.7. Cytoscape Visualization and Hub-Gene Ranking

The STRING-derived PPI network was exported and further analyzed using Cytoscape v3.10.4 for visualization and topological interrogation [21]. Network modules (highly interconnected clusters) were identified using the Molecular Complex Detection (MCODE) plugin with the following parameters: degree cutoff = 2, node score cutoff = 0.2, K-core = 2, and Max depth = 100, with cluster finding and haircut enabled. To prioritize hub genes within the PPI network, hub-gene ranking was performed using the cytoHubba plugin in Cytoscape. Three complementary algorithms—Degree, Density of Maximal Neighborhood Component (DMNC), and Edge Percolated Component (EPC)—were applied to compute hub scores, and the top 10 genes from each method were extracted. To obtain a robust hub-gene set and reduce algorithm-specific bias, the overlap among the three top-10 lists was determined using a Venn-diagram, and genes present in the intersection were defined as consensus hub genes for downstream analyses.

### 2.8. Protein-Level Validation of Hub Genes

To evaluate whether the identified hub genes exhibit consistent alterations at the protein level in pancreatic cancer, protein expression was examined using the University of Alabama at Birmingham Cancer Data Analysis Portal (UALCAN) (https://ualcan.path.uab.edu/, accessed on 10 February 2026) [22]. UALCAN provides an interface for proteomic analyses derived from the Clinical Proteomic Tumor Analysis Consortium (CPTAC) and the International Cancer Proteogenome Consortium (ICPC) datasets. For each hub gene, protein abundance was compared between primary tumor and normal pancreatic tissues to assess tumor-normal differences. In addition, protein expression was further stratified by pathological stage to investigate whether protein-level changes were associated with disease progression.

### 2.9. Survival Analysis of Hub Genes

The prognostic significance of prioritized hub-gene candidates was evaluated using Kaplan–Meier Plotter, which integrates gene expression and survival data across multiple cancer types. The present study focused on COL5A2 as the final consensus hub selected for downstream validation (https://kmplot.com/analysis/, accessed on 11 February 2026) [23]. Analyses were conducted under the pancreatic cancer mRNA-CHIP subsystem. Patients were stratified using trichotomization based on expression quartiles (Q1 vs. Q4), comparing the lowest-expression quartile (Q1) with the highest-expression quartile (Q4). The association between hub-gene expression and patient outcomes was evaluated using overall survival and disease-free survival endpoints; the corresponding hazard ratios and log-rank *p*-values were recorded for interpretation.

### 2.10. Receiver Operating Characteristic Analysis

To compare the tumor–non-tumor discriminatory performance of COL5A2 with related ECM-associated collagen genes, receiver operating characteristic (ROC) analyses were performed in the three bulk GEO cohorts (GSE15471, GSE16515, and GSE62452) for COL5A2, COL1A1, COL3A1, and COL5A1. For each dataset, gene-level expression values were obtained from the normalized expression matrix after probe-to-gene mapping using the corresponding GEO platform annotation. When multiple probes mapped to a given gene, probe-level values were averaged to obtain a single gene-level expression value per sample. Samples were classified as tumor or non-tumor according to dataset-specific GEO metadata parsing rules and validated against expected cohort counts. ROC curves were generated in R using the pROC package, and the area under the curve (AUC), 95% confidence interval, optimal cutoff (Youden index), sensitivity, and specificity were recorded. Pairwise DeLong tests were further applied to compare the ROC performance of COL5A2 against COL1A1, COL3A1, and COL5A1 within each cohort.

### 2.11. Early Progression Cohort Analysis

The GEO dataset GSE43288 was downloaded from GEO. Note that analyses involving COL5A2 were performed using the GPL96 platform because COL5A2 is not represented on GPL97. Samples were assigned to Normal, PanIN, or PDAC based on GEO sample identifiers and annotations, and technical replicate arrays from the same specimen were collapsed into a single value by taking the mean to define the patient-level analysis unit. CAF and ECM signature scores were computed using predefined gene sets (Supplementary Table S10). For each gene, expression was z-scored across samples, and the signature score for each sample was defined as the mean z-score across genes within the corresponding signature. Sample-level signature scores were then collapsed to the patient level by averaging across technical replicates. Group comparisons across the Normal, PanIN, and PDAC groups were performed using the Kruskal–Wallis test, followed by pairwise Wilcoxon tests with Benjamini–Hochberg adjustment where applicable. Progression trends across ordered groups (Normal = 0, PanIN = 1, and PDAC = 2) were evaluated using ordinary least squares slope, Spearman correlation, and the Jonckheere-Terpstra test. To directly assess the relationship between COL5A2 and stromal programs within the progression cohort, patient-level correlation analyses were performed between COL5A2 expression and ECM scores as well as between COL5A2 expression and CAF signature scores. Correlation strength and significance were evaluated using Spearman’s rank correlation. To further determine whether the association of COL5A2 with stromal programs was independent of disease group, we performed multivariable linear regression analyses in the patient-level GSE43288 cohort. The ECM score or CAF score was modeled as the dependent variable, COL5A2 expression as the predictor of interest, and disease group (Normal, PanIN, and PDAC) as the adjustment variable. In sensitivity analyses, disease progression was alternatively modeled as an ordinal stage variable (Normal = 0, PanIN = 1, and PDAC = 2). In addition, partial correlation analyses controlling for disease group were performed to further evaluate the adjusted association between COL5A2 expression and ECM/CAF signature scores. Statistical results from the adjusted regression, sensitivity, and partial-correlation analyses were exported as Supplementary Tables (Supplementary Tables S7–S9). Figures and statistical tables were generated using custom R scripts.

### 2.12. Single-Nucleus RNA Sequencing Dataset

The supplementary AnnData (.h5ad) object provided for GSE202051 was downloaded from GEO and imported into R. The treatment-naïve PDAC subset was reconstructed directly from the processed .h5ad metadata using the specimen field (sampleid), the treatment field (new treatment), and the cell-type annotation field (new_celltypes), thereby aligning the analysis with the original GEO study design (18 untreated PDAC specimens, 25 treated PDAC specimens, and 2 non-malignant specimens). Untreated PDAC specimens were retained by combining treatment-naïve annotations with a tumor-cell guard based on specimen-level cell-type composition. For overview analyses, nuclei were summarized into five major categories (Tumor, CAF, myCAF, Immune, and Other) to visualize specimen-level composition and the major-category contribution to total COL5A2 counts. For subtype-level quantification, fibroblast-lineage nuclei were further stratified into Fibroblast-CAF, Fibroblast-myCAF, Fibroblast-iCAF, Fibroblast-Pericyte, and Fibroblast-vSMC. We then calculated subtype composition within the fibroblast lineage, the fraction of fibroblast-associated COL5A2 signal contributed by each subtype, and per-specimen/per-nucleus COL5A2 summary metrics for each subtype. These outputs were exported for downstream figure preparation and summarized in Supplementary Table S1. Note that because the present study used the processed .h5ad object provided by the dataset, raw-level quality-control filtering, normalization, and upstream cell-calling procedures were not re-performed in the present study and were assumed to follow those of the original dataset resource.

### 2.13. Statistical Analysis

Differential expression results generated by GEO2R (limma) were interpreted using Benjamini–Hochberg FDR correction. Statistical significance was defined as adjusted *p*-value (FDR) < 0.01 with an absolute log_2_ fold change (|log_2_FC|) ≥ 1. For enrichment analyses, statistical significance was assessed using the multiple-testing–adjusted *p*-values reported by each platform. Cancer hallmark enrichment was interpreted based on the adjusted *p*-values provided by CancerHallmarks.com. GO/KEGG enrichment was performed in ShinyGO v0.85.1 using an FDR cutoff of 0.05 and a minimum pathway size of 10 (default). Protein-level comparisons queried from UALCAN (CPTAC/ICPC) and survival analyses conducted in Kaplan–Meier Plotter were reported according to the statistical procedures implemented by each web platform. The corresponding *p*-values, hazard ratios, confidence intervals, and log-rank statistics were recorded directly from the platform outputs.

For targeted expression comparisons derived from custom R scripts, non-parametric tests were applied when distributional assumptions could not be guaranteed. Multi-group comparisons were evaluated using the Kruskal–Wallis test followed by pairwise Wilcoxon tests with Benjamini–Hochberg correction where applicable. Correlation analyses between COL5A2 expression and ECM/CAF signature scores were evaluated using Spearman’s rank correlation. ROC analyses were summarized by AUC, 95% confidence interval, optimal cutoff, sensitivity, and specificity; pairwise ROC comparisons were evaluated using the DeLong test. All hypothesis tests were two-sided unless otherwise specified. For the GSE43288 adjusted analyses, multivariable linear regression and partial correlation controlling for disease group were used to evaluate whether associations between COL5A2 expression and ECM/CAF signature scores remained after accounting for progression group.

## 3. Results

### 3.1. Cross-Cohort Identification of Robust PDAC-Associated DEGs

**Figure 2:**
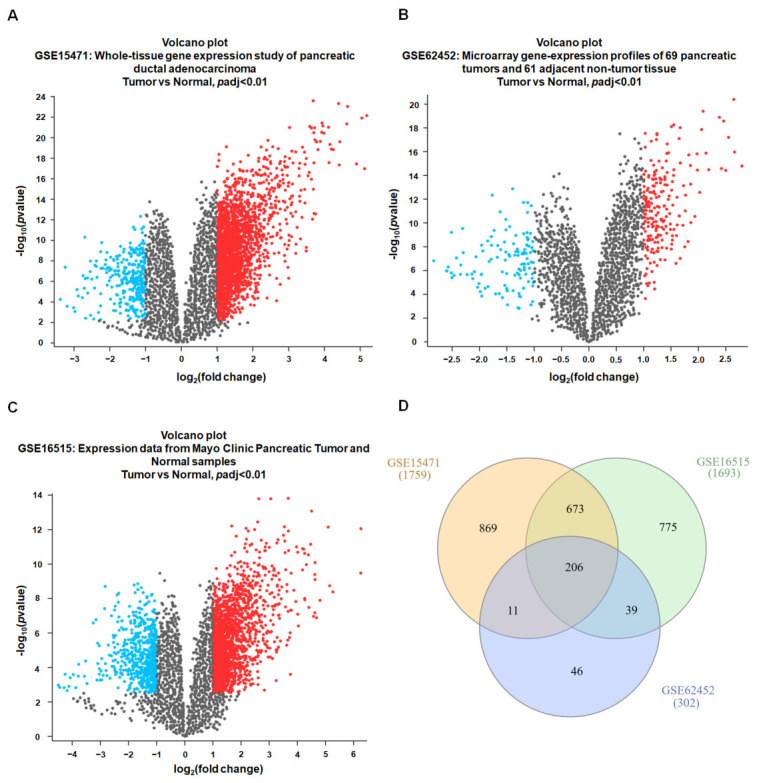
Identification of robust DEGs in PDAC across multiple GEO cohorts. (**A**–**C**) Volcano plots showing DEGs between PDAC tumor and normal pancreatic tissues in three independent GEO datasets (GSE15471, GSE62452, and GSE16515) analyzed using GEO2R (limma). Genes meeting the pre-specified criteria (|log_2_FC| ≥ 1 and adjusted *p* < 0.01; Benjamini–Hochberg correction) are highlighted as significant. Upregulated genes are shown in red, downregulated genes in blue, and non-significant genes in gray. (**D**) Venn diagram showing the overlap of significant DEGs among the three datasets. Different colors represent the three GEO datasets (GSE15471, GSE16515, and GSE62452), and the overlapping regions indicate shared DEGs. The numbers indicate the numbers of significant DEGs identified in each individual dataset and the numbers of common DEGs in the intersecting regions.

To identify genes consistently dysregulated in PDAC, we analyzed three independent microarray datasets from the GEO database (GSE15471, GSE62452, and GSE16515; Figure 2A–C). Samples were stratified into normal and tumor groups, and DEGs were identified using GEO2R with thresholds of |log_2_FC| ≥ 1 and adjusted *p* < 0.01. This analysis yielded 1759 DEGs in GSE15471, 1693 in GSE16515, and 302 in GSE62452. To evaluate reproducibility across datasets and platforms, we performed an intersection analysis using a Venn diagram and identified 206 DEGs consistently and significantly dysregulated across all three cohorts (Figure 2D). Collectively, these consensus DEGs represent a robust PDAC-associated transcriptional signature and were prioritized for downstream functional and network-based analyses.

### 3.2. Enrichment Profiling Links Shared PDAC DEGs to ECM Organization, Migration, and Focal Adhesion Signaling

**Figure 3:**
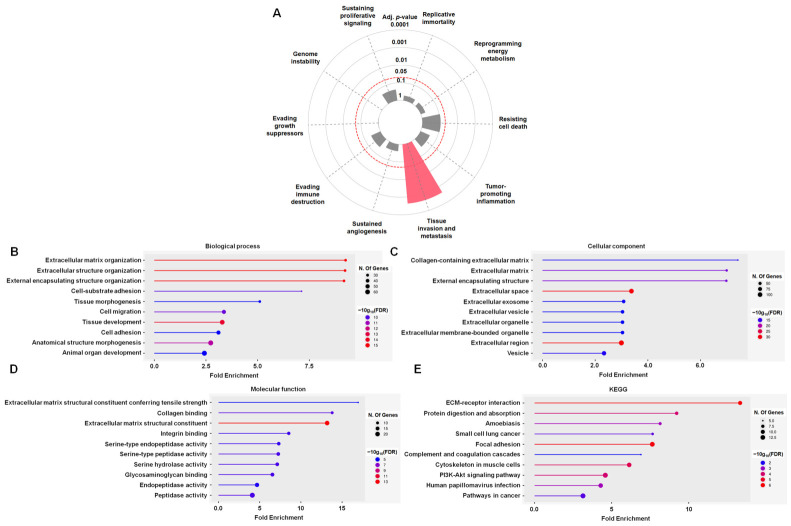
Functional enrichment analysis of the 206 DEGs highlights ECM-centered biology. (**A**) Hallmark enrichment plot generated using the Cancer Hallmarks Analytics Tool, showing significant hallmark terms enriched by the 206 shared DEGs (adjusted *p*-value thresholds as defined by the platform). Gray dashed lines delineate individual hallmark categories, and the red dashed line marks the adjusted *p*-value cutoff of 0.05. Hallmark categories with adjusted *p*-values greater than 0.05 are displayed in gray, whereas those with adjusted *p*-values less than 0.05 are displayed in color. Gray concentric circular lines indicate different adjusted *p*-value levels. (**B**–**D**) GO enrichment results produced with ShinyGO (v0.85.1), displaying the top 10 enriched terms for biological process, cellular component, and molecular function. (**E**) KEGG pathway enrichment analysis of the 206 shared DEGs. Bubble size indicates the number of genes mapped to each term; color indicates −log_10_ false discovery rate.

To characterize the biological relevance of the 206 consensus DEGs identified across the three PDAC cohorts, we performed pathway and functional enrichment analyses. Hallmark enrichment analysis indicated that these DEGs were significantly overrepresented in cancer-related programs associated with invasion and metastasis (adjusted *p* < 0.001; Figure 3A). We next conducted GO enrichment to delineate associated biological processes, cellular components, and molecular functions. In the biological process category, the DEGs were enriched for terms related to ECM remodeling and tissue architecture, including ECM organization, extracellular structure organization, cell–substrate adhesion, tissue morphogenesis, cell migration, and cell adhesion (Figure 3B). In the cellular component category, enriched terms were predominantly ECM- and extracellular compartment-related, such as collagen-containing ECM, the ECM itself, extracellular region/space, and vesicle-associated compartments (Figure 3C). In the molecular function category, enrichment was observed for ECM structural constituents and binding activities, including ECM structural constituent, collagen binding, integrin binding, and glycosaminoglycan binding, together with peptidase-related activities consistent with matrix remodeling (Figure 3D). Finally, KEGG pathway analysis further supported the predominance of ECM and adhesion signaling, highlighting ECM-receptor interaction, focal adhesion, PI3K-Akt signaling, complement and coagulation cascades, and broader cancer-related pathways (Figure 3E). Collectively, these enrichment results suggest that the common PDAC-associated transcriptional signature is strongly linked to ECM remodeling and cell–matrix interactions, processes that are broadly associated with tumor invasion, stromal remodeling, and microenvironmental reprogramming.

### 3.3. Network-Based Prioritization Reveals COL5A2 as the Top Consensus Hub Among Shared DEGs

**Figure 4:**
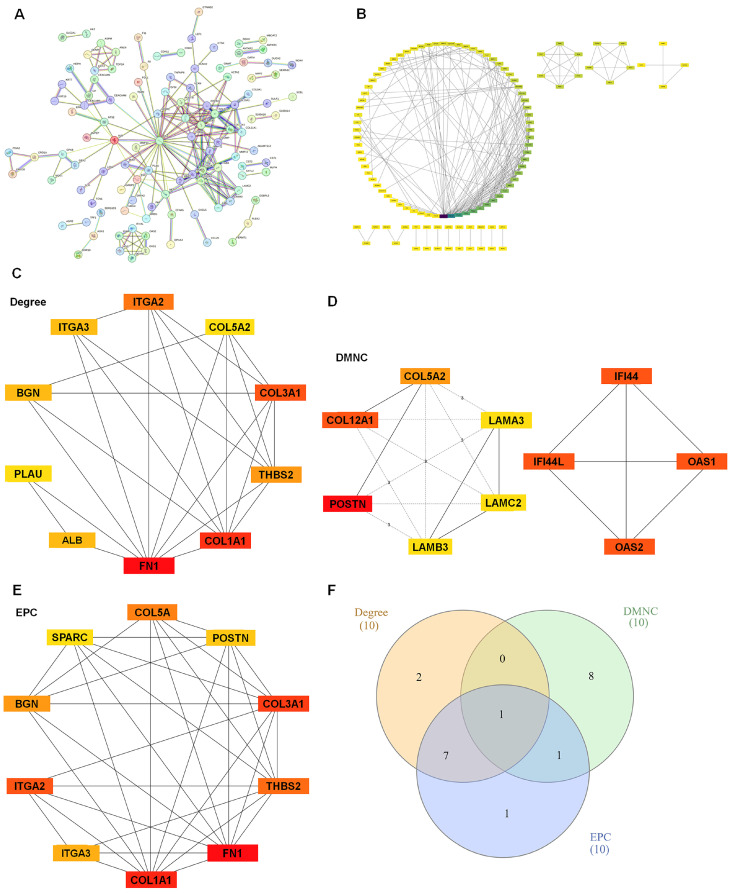
PPI network construction and hub-gene nomination identified COL5A2 as a consensus hub. (**A**) STRING PPI network constructed from the 206 shared DEGs (*Homo sapiens*) using a high-confidence interaction score threshold (combined score ≥ 0.7). The network contained 204 nodes and 209 edges, with an expected number of edges of 33, an average node degree of 2.05, an average local clustering coefficient of 0.391, and significant PPI enrichment (*p* < 1.0 × 10^−16^). Nodes represent proteins encoded by DEGs, and edges represent STRING-supported interactions. (**B**) Cytoscape-based module detection using MCODE, showing major network clusters derived from the PPI network. (**C**–**E**) Hub-gene ranking using cytoHubba under three complementary algorithms (Degree, DMNC, and EPC), showing the top 10-ranked genes identified by each method. In these panels, node colors indicate the ranking order assigned by each algorithm, with orange-red representing higher-ranked genes and yellow representing lower-ranked genes. Solid lines indicate direct interactions between nodes in the original network, whereas dashed lines indicate indirect connections between top-ranked nodes through intermediate nodes outside the displayed top-ranked set. The numbers labeled on dashed lines represent the shortest path distance between the two nodes in the original network. The DMNC-derived top-ranked genes formed two subnetworks in the reconstructed PPI network. (**F**) Venn diagram showing the overlap among the top-ranked candidates from the three hub-ranking methods. Different colors represent the different ranking methods, and the overlapping regions indicate shared hub-gene candidates identified by more than one method. The numbers indicate the numbers of candidate genes uniquely identified by each method and the numbers shared among methods.

To delineate functional relationships among the 206 consensus DEGs and prioritize key hub candidates, we constructed a PPI network using STRING. At a high-confidence interaction threshold (combined score ≥ 0.7), the resulting network comprised 204 nodes and 209 edges, with an expected number of edges of 33, an average node degree of 2.05, and an average local clustering coefficient of 0.391. The network also showed significant PPI enrichment (*p* < 1.0 × 10^−16^), indicating that these DEGs formed a biologically interconnected module rather than a random gene set (Figure 4A). We next imported the PPI network into Cytoscape to identify densely connected subnetworks. MCODE analysis partitioned the network into five major clusters (Figure 4B), suggesting distinct but related functional modules within the shared PDAC transcriptional signature. To further prioritize hub genes, we applied cytoHubba using three complementary algorithms (Degree, DMNC, and EPC) and extracted the top 10 genes from each ranking (Figure 4C–E). Intersection analysis across the three algorithms identified COL5A2 as the only gene consistently ranked as a hub by all methods (Figure 4F). Based on its reproducible network centrality across independent scoring strategies, COL5A2 was selected for subsequent validation analyses.

### 3.4. Proteomic Upregulation, Adverse Survival Association, and Tumor–Non-Tumor Discriminatory Performance of COL5A2 in PDAC

**Figure 5:**
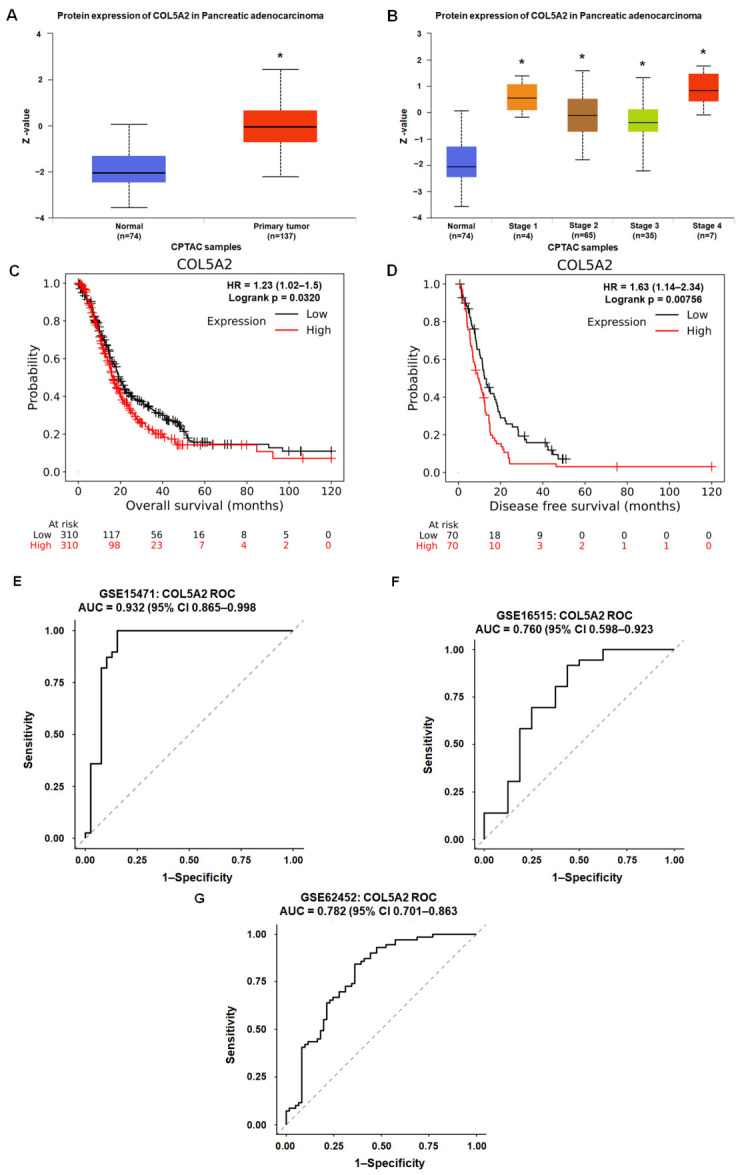
External validation of COL5A2 at the protein level, association with patient outcomes, and tumor–non-tumor discriminatory performance. (**A**) COL5A2 protein abundance in pancreatic adenocarcinoma versus normal tissue assessed using UALCAN based on CPTAC/ICPC proteomics resources. * *p* < 0.05 indicates significance. (**B**) Stage-stratified COL5A2 protein abundance across pancreatic adenocarcinoma stages compared with normal tissue (UALCAN; CPTAC/ICPC); * *p* < 0.05 indicates significance. (**C**,**D**) Kaplan–Meier survival analyses performed using Kaplan–Meier Plotter (mRNA-CHIP category), comparing high versus low COL5A2 expression groups using the Q1 vs. Q4 cutoff. Overall survival and disease-free survival are shown with hazard ratio (HR) and log-rank *p*-value provided by the platform. In the numbers shown below the plots, red indicates the number of patients in the high-COL5A2-expression group and black indicates the number of patients in the low-COL5A2-expression group remaining at risk at each time point. (**E**–**G**) ROC curves evaluating the tumor–non-tumor discriminatory performance of COL5A2 in GSE15471, GSE16515, and GSE62452. The area under the curve (AUC) is shown for each cohort.

To evaluate the clinical relevance of COL5A2 in PDAC, we next examined the expression of COL5A2 using publicly available proteomic and survival resources. Using UALCAN (CPTAC/ICPC-based proteomics), COL5A2 protein abundance was significantly higher in pancreatic adenocarcinoma than in normal pancreatic tissue (Figure 5A). Moreover, COL5A2 protein expression remained significantly elevated relative to normal tissue across all clinical stages (stage 1–4; Figure 5B), indicating stage-spanning upregulation at the protein level. We further assessed the survival association of COL5A2 using quartile-based stratification (Q1 vs. Q4) in the mRNA-CHIP database. Patients with higher COL5A2 expression exhibited significantly worse overall survival (HR = 1.23, log-rank *p* = 0.0320; Figure 5C) and disease-free survival (HR = 1.63, log-rank *p* = 7.56 × 10^−3^; Figure 5D). Head-to-head ROC analyses comparing COL5A2 with COL1A1, COL3A1, and COL5A1 across three independent bulk PDAC cohorts revealed that COL5A2 remained among the top-performing genes in each dataset (Figure 5E–G; Supplementary Table S2). In GSE15471, COL5A2 yielded an AUC of 0.932, which was comparable to COL5A1 (0.931) and slightly higher than COL3A1 (0.920) and COL1A1 (0.917). In GSE16515, COL5A2 showed the highest AUC (0.760), exceeding COL5A1 (0.726), COL3A1 (0.681), and COL1A1 (0.665). In GSE62452, COL5A2 again ranked highest (AUC 0.782), followed by COL5A1 (0.774), COL1A1 (0.751), and COL3A1 (0.735). DeLong testing identified that COL5A2 significantly outperformed COL3A1 in GSE16515 (*p* = 0.0020) and GSE62452 (*p* = 0.0011), whereas differences versus COL1A1 and COL5A1 were not significant. Collectively, these findings indicate that COL5A2 is consistently upregulated in PDAC, is associated with adverse survival outcomes, and demonstrates stable tumor-versus-non-tumor discriminatory performance across independent cohorts, supporting the prioritization of COL5A2 as a stromal-associated candidate for further biomarker evaluation.

### 3.5. Integrative Bulk and Single-Nucleus Analyses Link COL5A2 to Stromal Programs and Fibroblast-Lineage Contribution in PDAC

**Figure 6:**
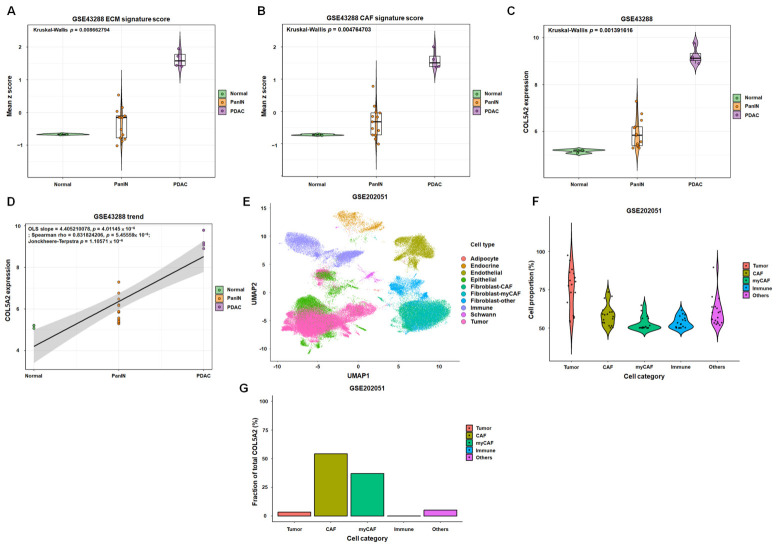
COL5A2 tracks stromal programs across early progression and is predominantly attributable to fibroblast-lineage compartments in untreated PDAC. (**A**) Comparison of ECM signature scores across the normal, PanIN, and PDAC groups in the early-progression cohort GSE43288 (GPL96). Scores were calculated by z-scoring signature genes across samples and summarizing each sample as the mean z-score across genes within the signature, followed by patient-level collapsing to account for technical replicate arrays. Violin plots show the distribution of patient-level values with overlaid points representing individual patients. (**B**) Comparison of CAF signature score across the normal, PanIN, and PDAC groups in GSE43288 (GPL96), computed and visualized as in (**A**). (**C**) Comparison of COL5A2 expression (log_2_ microarray expression; GPL96) across the normal, PanIN, and PDAC groups in GSE43288 after patient-level collapsing. (**D**) Ordered-progression trend analysis of COL5A2 across the normal-PanIN-PDAC continuum in GSE43288. The trend is summarized by the ordinary least squares slope and supported by non-parametric monotonic association tests (Spearman correlation and Jonckheere-Terpstra test). Colored points represent individual samples from the different disease groups, the black line indicates the ordinary least squares (OLS) trend line, and the gray shaded band represents the confidence interval around the fitted trend. (**E**) Uniform Manifold Approximation and Projection visualization of nuclei from untreated PDAC specimens in GSE202051 using the dataset-provided embedding. Cell-type labels were consolidated into broader categories to simplify visualization. (**F**) Specimen-level cellular composition in untreated PDAC (GSE202051). Nuclei proportions were summarized per specimen into five major categories (Tumor, CAF, myCAF, Immune, and Other) and visualized using violin plots with overlaid specimen points. (**G**) COL5A2 contribution by major cell category in untreated PDAC (GSE202051). COL5A2 contribution was computed from raw counts (AnnData layer “counts”; if unavailable, the primary expression matrix X was used) by summing COL5A2 counts within each major category and calculating the fraction of total COL5A2 counts attributable to that category. Colors in the relevant panels are used solely to distinguish sample groups or cell categories. In the specimen-level composition plot, categories with very low abundance may appear as minimal or not readily visible segments because of their small proportional contribution.

Because COL5A2 encodes the alpha2(V) chain of type V collagen, a regulatory fibrillar collagen associated with ECM organization [24,25,26], we next examined whether COL5A2 tracked stromal programs during early pancreatic tumorigenesis and whether its expression could be attributed to fibroblast-lineage compartments. Thus, we analyzed the microarray dataset GSE43288, which includes samples spanning normal pancreas, PanIN, and PDAC. PanIN provides a biologically relevant intermediate state for evaluating progression-associated molecular changes. Using predefined stromal signatures (Supplementary Table S10), we quantified ECM and CAF scores across samples and observed significant differences among the three pathological groups (ECM: Kruskal–Wallis *p* = 8.7 × 10^−3^; CAF: Kruskal–Wallis *p* = 4.8 × 10^−3^; Figure 6A,B; Supplementary Table S3). Detailed pairwise group comparison statistics for ECM score, CAF score, and COL5A2 expression across the Normal, PanIN, and PDAC groups are provided in Supplementary Table S4. Consistently, COL5A2 expression also differed significantly across normal, PanIN, and PDAC groups (Kruskal–Wallis *p* = 1.4 × 10^−3^; Figure 6C; Supplementary Table S3). We next evaluated progression-associated trends across the ordered groups (normal, PanIN, and PDAC). COL5A2 exhibited a strong increasing trend across the progression continuum, supported by concordant results from multiple complementary tests (ordinary least squares slope = 4.41, *p* = 4.01 × 10^−6^; Spearman ρ = 0.832, *p* = 5.46 × 10^−6^; Jonckheere-Terpstra *p* = 1.11 × 10^−6^; Figure 6D; Supplementary Table S5). In addition to this ordered progression pattern, patient-level correlation analyses revealed that COL5A2 expression was strongly correlated with both the ECM signature score (Spearman ρ = 0.875, *p* = 4.40 × 10^−7^) and the CAF signature score (Spearman ρ = 0.938, *p* = 9.76 × 10^−10^), further supporting a close association between COL5A2 and stromal programs in the progression cohort (Supplementary Figure S1; Supplementary Table S6). As COL5A2, ECM score, and CAF score all increased along the normal-PanIN-PDAC axis, we next examined whether these associations remained significant after adjustment for disease group. In multivariable linear regression analyses after adjustment for disease group, COL5A2 expression remained significantly associated with ECM score (beta = 0.364, *p* < 0.001; Supplementary Table S7 and Supplementary Figure S2A) and CAF score (beta = 0.393, *p* < 0.001; Supplementary Table S7 and Supplementary Figure S2B). Sensitivity analyses using ordinal stage coding instead of categorical disease group yielded consistent results (Supplementary Table S8), and group-adjusted partial correlation analyses further supported the robustness of these relationships (Supplementary Table S9). Together, these findings indicate that higher ECM/CAF signature scores and higher COL5A2 expression co-occur along the normal-PanIN-PDAC axis in bulk tissue profiles and that the association between COL5A2 and stromal signature activity cannot solely be explained by progression group.

Given that bulk transcriptomic profiles reflect mixed cell populations, we next evaluated the cellular context of COL5A2 using the single-nucleus RNA sequencing (snRNA-seq) dataset GSE202051. Reanalysis of the dataset-provided .h5ad object confirmed that the treatment-naïve PDAC subset consisted of 18 specimens, consistent with the original GEO study design. The selected untreated PDAC specimens included in this reanalysis are listed in Supplementary Table S11. Uniform Manifold Approximation and Projection embedding was used to visualize major cell populations within these untreated PDAC tumor specimens (Figure 6E). Major-category summaries revealed that CAF- and myCAF-lineage populations together constituted a substantial fraction of the untreated PDAC microenvironment and accounted for the majority of the total COL5A2 signal, whereas tumor and immune compartments contributed comparatively smaller fractions (Figure 6F,G; Supplementary Table S12). We then resolved the fibroblast lineage into five subtypes and observed that Fibroblast-CAF represented the quantitatively dominant subtype (mean proportion within the fibroblast lineage = 0.5497) and the largest overall contributor to fibroblast-associated COL5A2 signal (mean contribution = 0.5570; Supplementary Table S1). In contrast, Fibroblast-myCAF showed the strongest COL5A2 enrichment at the per-specimen level (mean expression = 2.8445; mean COL5A2-positive fraction = 0.9249), whereas Fibroblast-iCAF, Fibroblast-Pericyte, and Fibroblast-vSMC exhibited lower overall representation and/or lower aggregate COL5A2 contribution (Supplementary Table S1). These subtype-level quantitative results support the interpretation that COL5A2 is predominantly derived from stromal/fibroblast compartments, with CAF and myCAF representing the principal sources of the signal in untreated PDAC.

## 4. Discussion

As the predominant histological subtype of pancreatic cancer, PDAC is characterized by a dense desmoplastic tumor microenvironment in which CAFs and ECM remodeling not only constitute a substantial fraction of tumor mass but also shape disease behavior. Although multiple stromal signatures and ECM-associated markers have been reported in PDAC, the specific role of COL5A2 in PDAC remains insufficiently defined, particularly in relation to cross-cohort robustness, progression-associated stromal remodeling, and fibroblast-lineage attribution at single-nucleus resolution. Here, we used a multi-cohort and multi-level strategy—spanning bulk transcriptomic screening, functional enrichment, network-based prioritization, external protein and survival resources, an early progression cohort (normal/PanIN/PDAC), and single-nucleus evidence—to evaluate COL5A2 as a reproducible, prioritized candidate associated with stromal/ECM features and PDAC progression.

A major methodological strength of this work was the emphasis on cross-cohort robustness. By applying uniform GEO2R/limma criteria with Benjamini–Hochberg FDR correction across three independent GEO cohorts (GSE15471, GSE62452, and GSE16515) and subsequently intersecting DEG lists, we enriched for signals consistently dysregulated across heterogeneous datasets and array platforms, thereby reducing the likelihood that downstream findings merely reflect cohort-specific technical or sampling artifacts. Although this “intersection-first” approach is conservative and could exclude biologically relevant but cohort-restricted signals, the resulting 206 shared DEGs formed a coherent ECM-centric signature, with enrichment for ECM organization, adhesion and migration programs, collagen-containing ECM components, and ECM-receptor interaction/focal adhesion pathways (Figure 3). This pattern aligns with prior PDAC transcriptomic meta-analyses and reviews that highlight stromal/ECM programs as a dominant axis of biological heterogeneity and clinical behavior [27].

Network-based analyses further supported COL5A2 as a reproducibly crucial component of this ECM/stromal axis. Using STRING v12.0 at a high-confidence interaction threshold (0.7) followed by Cytoscape-based module discovery (MCODE) and hub ranking (cytoHubba Degree/DMNC/EPC), COL5A2 was uniquely retained across multiple hub-ranking schemes (Figure 4). While topological centrality alone does not establish causality, consensus identification across independent algorithms strengthens our confidence that COL5A2 resides within a core ECM/adhesion module rather than being an artifact of a single analytic choice. Fibrillar collagens and stromal gene signatures have been broadly implicated in PDAC prognosis and therapeutic response; however, COL5A2 itself has been less directly emphasized and remains insufficiently characterized in PDAC. In this context, our findings support COL5A2 as an underexplored but biologically plausible candidate that warrants further evaluation within PDAC-specific stromal and ECM remodeling frameworks.

We next examined whether this transcriptomic candidate exhibits concordant patterns in independent external resources. In UALCAN proteomics (CPTAC/ICPC), COL5A2 protein abundance was higher in pancreatic adenocarcinoma than in normal tissue and remained elevated across disease stages (Figure 5A,B). Kaplan–Meier Plotter survival analyses (mRNA-CHIP category; Q1 vs. Q4 cutoff) further indicated that higher COL5A2 expression was associated with shorter overall survival and disease-free survival (Figure 5C,D). These platform-derived associations support the clinical relevance of COL5A2 as an ECM/stroma-associated candidate marker. It should be noted that these findings could still be influenced by cohort heterogeneity, tumor purity, and stromal fraction. Accordingly, independent validation in well-annotated cohorts and multivariable models will be necessary before concluding that COL5A2 provides prognostic information beyond established clinicopathologic covariates. Despite extensive literature on ECM remodeling and CAF biology in PDAC, relatively few studies have directly focused on COL5A2 in this disease context to date [28,29], further emphasizing COL5A2 as a potential biomarker candidate warranting more targeted evaluation. The novelty of the present study lies not simply in re-identifying an ECM-associated collagen gene but also in reproducibly prioritizing COL5A2 across independent bulk cohorts, demonstrating its stable tumor-versus-non-tumor discriminatory performance relative to related collagen markers, linking it to progression-associated stromal/ECM programs, and further localizing its expression to fibroblast-lineage compartments at the single-nucleus level.

Given that COL5A2 encodes the α2 chain of type V collagen, which is an ECM component enriched in stromal compartments, our observations are biologically probable within the broader context of desmoplastic PDAC. Collagen type V has been implicated as a regulatory fibrillar collagen that can modulate matrix architecture and cell–matrix interactions. Notably, experimental work has shown that collagen type V produced by pancreatic stellate cells can influence malignant phenotypes through β1-integrin–linked signaling and that suppressing stromal collagen V reduces invasion/metastasis in PDAC models [30]. More generally, CAF heterogeneity and ECM-producing fibroblast states have been repeatedly described in PDAC by single-cell studies, including multiple fibroblast programs with distinct immune and malignant interactions [31,32,33,34]. Taken together, these lines of evidence support the interpretation that COL5A2 in bulk PDAC profiles is closely linked to fibroblast/ECM programs, while still leaving open whether COL5A2 primarily reflects stromal abundance or functions as an active contributor to tumor-promoting matrix remodeling.

We therefore further tested whether COL5A2 tracked with early lesion progression and stromal program enrichment. In the early progression cohort GSE43288 (GPL96), ECM and CAF signature scores differed across the normal, PanIN, and PDAC groups; COL5A2 simultaneously showed a concordant increase along the normal-PanIN-PDAC spectrum (Figure 6A–D). Patient-level correlation analyses further revealed strong positive associations between COL5A2 expression and ECM signature scores as well as between COL5A2 expression and CAF signature scores (Supplementary Figure S1A,B; Supplementary Table S6). These results support COL5A2 as tightly coupled to desmoplastic stromal programs during PDAC progression rather than representing an isolated transcriptomic event [27]. Importantly, these associations remained significant after adjustment for disease group and were further supported by ordinal-stage sensitivity and partial-correlation analyses, indicating that the relationship between COL5A2 and stromal signature activity cannot be solely explained by progression-group differences.

Finally, single-nucleus evidence from GSE202051 provided cellular context for the bulk findings. By reconstructing the untreated PDAC subset directly from the processed .h5ad metadata provided by the dataset, we restricted downstream analyses to the 18 treatment-naive PDAC specimens defined by the original GEO study design. Major-category summaries revealed that COL5A2 expression was concentrated in fibroblast-lineage compartments. Subtype-level quantification further indicated that Fibroblast-CAF was the dominant contributor to total fibroblast-associated COL5A2 signal, whereas Fibroblast-myCAF showed the strongest per-specimen COL5A2 enrichment (Supplementary Table S1). These pieces of evidence—bulk progression analyses, adjusted and unadjusted associations with ECM/CAF programs, and single-nucleus cellular attribution—support COL5A2 as a primary stromal/fibroblast-derived marker linked to ECM remodeling and PDAC progression.

It should be noted that several limitations warrant consideration. First, cross-cohort DEG intersection enriches for robust signals but possibly excludes cohort-specific biology relevant to molecular subtypes, treatment status, or sampling differences. Second, network-based hub nomination is constrained by existing interaction knowledge and does not demonstrate causality. Third, the early progression cohort (GSE43288) has a modest sample size; although patient-level collapsing minimizes technical replication bias, replication in additional progression series and in prospective samples (e.g., PanIN or intraductal papillary mucinous neoplasms with long-term follow-up) will be important to confirm generalizability. Fourth, survival and proteomic associations derived from UALCAN and Kaplan–Meier Plotter are subject to cohort heterogeneity and incomplete adjustment for confounders such as stromal proportion, treatment regimens, and comorbidities. Finally, while GSE202051 supports a fibroblast/CAF-dominant origin of COL5A2, further work integrating finer-grained CAF substate annotations and spatially resolved technologies (e.g., spatial transcriptomics or multiplex imaging) will be necessary to define COL5A2-enriched niches and to understand how they interface with malignant, immune, and vascular compartments.

Despite these caveats, our integrative analysis suggests several directions for future investigation. First, COL5A2 could be evaluated as part of composite stromal/ECM signatures to refine risk stratification or to identify patients most likely to benefit from stroma-modulating or mechanotransduction-targeting therapies. Second, mechanistic studies—such as COL5A2 knockdown or overexpression in CAFs and co-culture or organoid models—could clarify whether COL5A2 actively modulates matrix architecture, stiffness, or integrin signaling that affect tumor cell invasion, immune exclusion, or therapy response. Third, combining COL5A2 assessment with CAF-subtype markers (e.g., myCAF, iCAF, or antigen-presenting CAF markers) in tissue microarrays or spatial platforms could elucidate whether COL5A2 is enriched in specific CAF subsets linked to distinct clinical trajectories or treatment sensitivities. Finally, it will be critical to determine whether COL5A2-associated stromal states are conserved across metastatic sites and treatment lines, which could inform the timing and context of potential COL5A2- or collagen V-targeted interventions.

## 5. Conclusions

Collectively, these multi-level findings support COL5A2 as a biologically potent stromal/ECM-associated candidate biomarker in PDAC. The integrated evidence indicates that COL5A2 is closely linked to progression-related stromal remodeling and is predominantly derived from stromal/fibroblast compartments, particularly CAF and myCAF populations. Further prospective validation and mechanistic investigation are warranted to clarify its clinical utility and functional role in PDAC progression.

## Data Availability

Publicly available datasets analyzed in this study are available from the NCBI Gene Expression Omnibus (GEO) under accession numbers GSE15471, GSE62452, GSE16515, GSE43288, and GSE202051. Proteomics results were queried via UALCAN (CPTAC/ICPC resources), and survival analyses were performed using the Kaplan–Meier Plotter web tool (pancreatic cancer; mRNA-CHIP category). The analysis scripts used to generate the figures and statistical outputs are available from the corresponding author upon reasonable request.

## Supplementary Materials

The following supporting information can be downloaded at: https://www.mdpi.com/article/10.3390/diagnostics16081205/s1. Supplementary Figure S1: Correlation of COL5A2 expression with ECM and CAF signature scores in GSE43288. (A) Correlation between COL5A2 expression and ECM signature score. (B) Correlation between COL5A2 expression and CAF signature score. Correlation coefficients and *p* values were calculated using Spearman correlation at the patient level after collapsing technical replicate arrays; Supplementary Figure S2: Adjusted association of COL5A2 expression with ECM and CAF signature scores after disease-group adjustment in GSE43288. (A) Adjusted residual plot for the association between COL5A2 expression and ECM signature score. (B) Adjusted residual plot for the association between COL5A2 expression and CAF signature score. Regression coefficients and *p* values were calculated using multivariable linear regression at the patient level after collapsing technical replicate arrays, with disease group included as an adjustment variable; Supplementary Table S1: Fibroblast subtype composition and COL5A2-associated quantitative metrics in the untreated PDAC subset of GSE202051; Supplementary Table S2: Head-to-head ROC comparison of COL5A2, COL1A1, COL3A1, and COL5A1 across three GEO PDAC cohorts; Supplementary Table S3: Overall group comparison statistics for ECM score, CAF score, and COL5A2 expression across the Normal, PanIN, and PDAC groups in the GSE43288 patient-level cohort; Supplementary Table S4: Pairwise group comparison statistics for ECM score, CAF score, and COL5A2 expression across the Normal, PanIN, and PDAC groups in the GSE43288 patient-level cohort; Supplementary Table S5: Progression trend statistics for ECM score, CAF score, and COL5A2 expression across the ordered Normal-PanIN-PDAC sequence in the GSE43288 patient-level cohort; Supplementary Table S6: Correlations between COL5A2 expression and ECM/CAF signature scores in GSE43288; Supplementary Table S7: Multivariable linear regression analyses of ECM and CAF scores adjusted for disease group; Supplementary Table S8: Sensitivity analyses using ordinal stage adjustment in GSE43288; Supplementary Table S9: Partial correlation analyses controlling for disease group in GSE43288; Supplementary Table S10: Definition of ECM and CAF signature gene sets used for score calculation; Supplementary Table S11: Selected untreated PDAC specimens included in the GSE202051 single-nucleus RNA sequencing reanalysis; and Supplementary Table S12: Major-category cell composition and COL5A2 contribution in untreated PDAC specimens from the GSE202051 single-nucleus RNA sequencing cohort.

## Abbreviations

The following abbreviations are used in this manuscript:

AnnData	annotated data matrix
BH	Benjamini–Hochberg
CAF	cancer-associated fibroblast
COL5A2	type V collagen alpha 2 chain
CPTAC	Clinical Proteomic Tumor Analysis Consortium
DEG	differentially expressed gene
DFS	disease-free survival
DMNC	density of maximal neighborhood component
ECM	extracellular matrix
EPC	edge percolated component
FDR	false discovery rate
FC	fold change
GEO	Gene Expression Omnibus
GO	Gene Ontology
ICPC	International Cancer Proteogenome Consortium
KEGG	Kyoto Encyclopedia of Genes and Genomes
limma	Linear Models for Microarray Data
MCODE	Molecular Complex Detection
myCAF	myofibroblastic cancer-associated fibroblast
OLS	ordinary least squares
OS	overall survival
PanIN	pancreatic intraepithelial neoplasia
PDAC	pancreatic ductal adenocarcinoma
PPI	protein–protein interaction
snRNA-seq	single-nucleus RNA-seq
STRING	Search Tool for the Retrieval of Interacting Genes/Proteins
TME	tumor microenvironment
UMAP	Uniform Manifold Approximation and Projection
