# Supplementary material for: Integrative Bulk and Single-Nucleus Analyses Nominate COL5A2 as a CAF/ECM-Associated Marker Associated with PDAC Progression
Source: Diagnostics (Basel). 2026 Apr 17;16(8):1205. doi: 10.3390/diagnostics16081205 (PMC13115138; doi:10.3390/diagnostics16081205)
Supplement: Supplementary file 1 [file diagnostics-16-01205-s001.zip › Supplemental Figures.pdf]

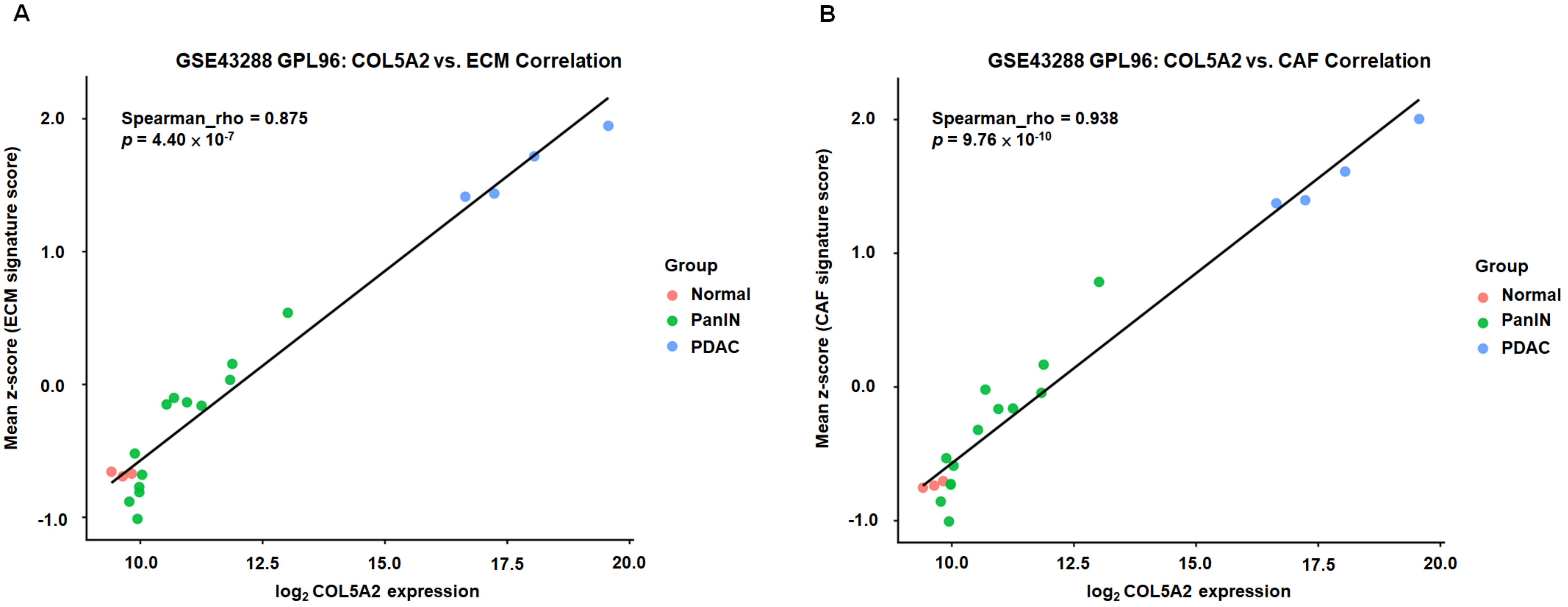

A
GSE43288 GPL96: COL5A2 vs. ECM Correlation
Spearman_rho = 0.875
p = 4.40 x 10-7
Mean z-score (ECM signature score)
2.0
1.0
0.0
-1.0
10.0
12.5
15.0
17.5
20.0
log2 COL5A2 expression
Group
Normal
PanIN
PDAC
B
GSE43288 GPL96: COL5A2 vs. CAF Correlation
Spearman_rho = 0.938
p = 9.76 x 10-10
Mean z-score (CAF signature score)
2.0
1.0
0.0
-1.0
10.0
12.5
15.0
17.5
20.0
log2 COL5A2 expression
Group
Normal
PanIN
PDAC

Supplementary Figure S1. Correlation of COL5A2 expression with ECM and CAF signature scores in GSE43288. (A) Correlation between COL5A2 expression and ECM signature score. (B) Correlation between COL5A2 expression and CAF signature score. Correlation coefficients and *p* values were calculated using Spearman correlation at the patient level after collapsing technical replicate arrays.

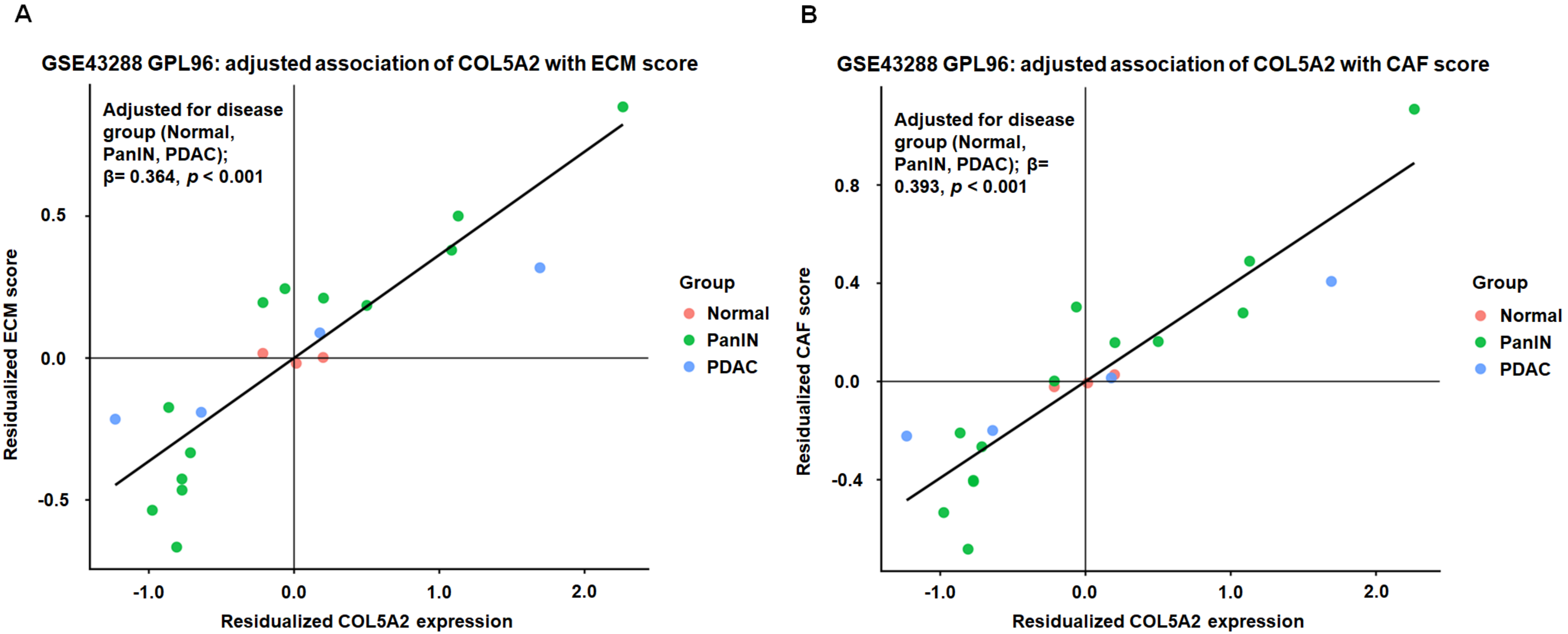
A
GSE43288 GPL96: adjusted association of COL5A2 with ECM score
Adjusted for disease group (Normal, PanIN, PDAC); β= 0.364, p < 0.001
0.5
0.0
-0.5
Residualized ECM score
-1.0
0.0
1.0
2.0
Residualized COL5A2 expression
Group
Normal
PanIN
PDAC
B
GSE43288 GPL96: adjusted association of COL5A2 with CAF score
Adjusted for disease group (Normal, PanIN, PDAC); β= 0.393, p < 0.001
0.8
0.4
0.0
-0.4
Residualized CAF score
-1.0
0.0
1.0
2.0
Residualized COL5A2 expression
Group
Normal
PanIN
PDAC

Supplementary Figure S2. Adjusted residual plots of COL5A2 expression with ECM and CAF signature scores in GSE43288. (A) Adjusted residual plot for the association between COL5A2 expression and ECM signature score. **(B)** Adjusted residual plot for the association between COL5A2 expression and CAF signature score. Regression coefficients and *p* values were calculated using multivariable linear regression at the patient level after collapsing technical replicate arrays, with disease group included as an adjustment variable.
