# Supplementary material for: Integrative Bulk and Single-Nucleus Analyses Nominate COL5A2 as a CAF/ECM-Associated Marker Associated with PDAC Progression
Source: Diagnostics (Basel). 2026 Apr 17;16(8):1205. doi: 10.3390/diagnostics16081205 (PMC13115138; doi:10.3390/diagnostics16081205)
Supplement: Supplementary file 1 [file diagnostics-16-01205-s001.zip › Supplementary tables.pdf]

Table 1. Fibroblast subtype composition and COL5A2-associated quantitative metrics in the untreated PDAC subset of GSE202051.

Fibroblast subtype	Specimens detected (n)	Mean proportion within fibroblast lineage	Median proportion within fibroblast lineage	Specimens detected for contribution (n)	Mean COL5A2 contribution within fibroblast lineage	Median COL5A2 contribution within fibroblast lineage	Per-specimen subtype observations (n)	Mean nuclei per specimen	Mean per-specimen COL5A2 expression	Mean COL5A2-positive fraction
Fibroblast-CAF	18.0000	0.5497	0.5240	18.0000	0.5570	0.4793	18.0000	638.2778	2.1525	0.7357
Fibroblast-myCAF	18.0000	0.2340	0.0592	18.0000	0.2636	0.0781	16.0000	430.0625	2.8445	0.9249
Fibroblast-iCAF	16.0000	0.0736	0.0306	16.0000	0.0705	0.0232	11.0000	219.6364	2.1404	0.7816
Fibroblast-Pericyte	18.0000	0.1036	0.0796	18.0000	0.0762	0.0509	17.0000	114.2353	1.6692	0.6293
Fibroblast-vSMC	6.0000	0.1416	0.0203	6.0000	0.1215	0.0165	3.0000	816.3333	1.8382	0.7898

Values summarize subtype composition, subtype-level contribution to fibroblast-associated COL5A2 signal, and per-specimen COL5A2-related metrics across the untreated PDAC subset.

Table 2. Head-to-head ROC comparison of COL5A2, COL1A1, COL3A1, and COL5A1 across three GEO PDAC cohorts.

Dataset	Gene	Tumor samples (n)	Non-tumor samples (n)	AUC	95% CI	Sensitivity	Specificity	DeLong *p* value vs COL5A2
GSE15471	COL5A2	39	39	0.9316	0.865-0.998	1.0000	0.8462	Reference
GSE15471	COL1A1	39	39	0.9165	0.844-0.989	1.0000	0.8205	0.1283
GSE15471	COL3A1	39	39	0.9198	0.849-0.991	1.0000	0.8462	0.3290
GSE15471	COL5A1	39	39	0.9310	0.872-0.990	0.9744	0.8205	0.9508
GSE16515	COL5A2	36	16	0.7604	0.598-0.923	0.9167	0.5625	Reference
GSE16515	COL1A1	36	16	0.6649	0.474-0.856	0.9167	0.5000	0.0524
GSE16515	COL3A1	36	16	0.6806	0.490-0.871	0.9722	0.4375	0.0020
GSE16515	COL5A1	36	16	0.7257	0.552-0.900	1.0000	0.4375	0.0775
GSE62452	COL5A2	69	61	0.7819	0.701-0.863	0.8406	0.6393	Reference
GSE62452	COL1A1	69	61	0.7508	0.663-0.838	0.9565	0.4918	0.0898
GSE62452	COL3A1	69	61	0.7349	0.645-0.825	0.9275	0.5246	0.0011
GSE62452	COL5A1	69	61	0.7736	0.691-0.856	0.9130	0.5410	0.6233

AUC, 95% confidence interval, sensitivity, and specificity are shown for each gene within each cohort. Pairwise ROC comparisons versus COL5A2 were evaluated using the DeLong test.

Table 3. Overall group comparison statistics for ECM score, CAF score, and COL5A2 expression across the Normal, PanIN, and PDAC groups in the GSE43288 patient-level cohort.

metric	Kruskal–Wallis *p*
ECM score	8.7×10^{-3}
CAF score	4.8×10^{-3}
COL5A2 $\log_2$	1.4×10^{-3}

Overall group differences were assessed using the Kruskal–Wallis test at the patient level after collapsing technical replicate arrays.

Table 4. Pairwise group comparison statistics for ECM score, CAF score, and COL5A2 expression across the Normal, PanIN, and PDAC groups in the GSE43288 patient-level cohort.

Metric	Comparison	*p* adj BH
ECM score	Normal vs PanIN	0.5012
ECM score	Normal vs PDAC	7.77×10^{-2}
ECM score	PanIN vs PDAC	1.17×10^{-2}
CAF score	Normal vs PanIN	0.1389
CAF score	Normal vs PDAC	7.77×10^{-2}
CAF score	PanIN vs PDAC	1.17×10^{-2}
COL5A2 $\log_2$	Normal vs PanIN	2.32×10^{-2}
COL5A2 $\log_2$	Normal vs PDAC	5.18×10^{-2}
COL5A2 $\log_2$	PanIN vs PDAC	1.17×10^{-2}

Pairwise comparisons were performed using the Wilcoxon rank-sum test with Benjamini–Hochberg adjustment for multiple testing. Analyses were conducted at the patient level after collapsing technical replicate arrays.

Table 5. Progression trend statistics for ECM score, CAF score, and COL5A2 expression across the ordered Normal–PanIN–PDAC sequence in the GSE43288 patient-level cohort.

Metric	OLS slope	OLS *p*	Spearman rho	Spearman *p*	Jonckheere Terpstra *p*
ECM score	1.2282	2.22×10^{-5}	0.647	2.00×10^{-3}	1.3×10^{-3}
CAF score	1.2347	1.76×10^{-5}	0.7323	2.42×10^{-4}	1.24×10^{-4}
COL5A2_$\log_2$	4.4052	4.01×10^{-6}	0.8318	5.46×10^{-6}	1.11×10^{-6}

Progression trends were evaluated using ordinary least squares slope, Spearman correlation, and the Jonckheere–Terpstra test, with ordered group coding defined as Normal = 0, PanIN = 1, and PDAC = 2. Analyses were conducted at the patient level after collapsing technical replicate arrays.

Table 6. Correlations between COL5A2 expression and ECM/CAF signature scores in GSE43288.

x	y	n	Spearman rho	Spearman *p*	Pearson r	Pearson *p*
COL5A2_$\log_2$	ECM score	20	0.8752	4.4×10^{-7}	0.9783	9.4×10^{-14}
COL5A2_$\log_2$	CAF score	20	0.9383	9.76×10^{-10}	0.9758	2.45×10^{-13}

Correlations were calculated at the patient level after collapsing technical replicate arrays.

Table 7. Multivariable linear regression analyses of ECM and CAF scores adjusted for disease group.

Outcome	Adjustment model	Predictor	Beta coefficient	*p* value	95% CI	n	Model *p* value
ECM score	Adjusted for disease group	COL5A2 expression	0.364	<0.001	0.265-0.463	20	9.26×10^{-12}
CAF score	Adjusted for disease group	COL5A2 expression	0.393	<0.001	0.299-0.487	20	4.42×10^{-12}

https://doi.org/10.3390/xxxxx

For each outcome, ECM score or CAF score was modeled as the dependent variable, COL5A2 expression as the predictor of interest, and disease group as the adjustment variable.

Table 8. Sensitivity analyses using ordinal stage adjustment in GSE43288.

Outcome	Sensitivity model	Term	Beta coefficient	Standard error	t statistic	*p* value	95% CI
ECM score	Adjusted for ordinal stage	COL5A2 expression	0.301	0.026	11.362	<0.001	0.245-0.357
ECM score	Adjusted for ordinal stage	Ordinal stage	-0.097	0.139	-0.700	0.493	-0.391-0.196
CAF score	Adjusted for ordinal stage	COL5A2 expression	0.293	0.028	10.425	<0.001	0.234-0.353
CAF score	Adjusted for ordinal stage	Ordinal stage	-0.058	0.148	-0.394	0.698	-0.371-0.254

Sensitivity analyses modeled disease progression as an ordinal stage variable (Normal = 0, PanIN = 1, PDAC = 2).

Table 9. Partial correlation analyses controlling for disease group in GSE43288.

Outcome	Adjustment variable	n	Partial Spearman rho	Partial Spearman p value	Partial Pearson r	Partial Pearson p value
ECM score	Disease group	20	0.880	<0.001	0.889	<0.001
CAF score	Disease group	20	0.916	<0.001	0.911	<0.001

Partial correlations were calculated after adjustment for disease group.

Table 10. Definition of ECM and CAF signature gene sets used for score calculation.

Signature	Gene
ECM	COL1A1
ECM	COL1A2
ECM	COL3A1
ECM	COL5A2
ECM	COL6A1
ECM	COL6A2
ECM	FN1
ECM	SPARC
ECM	DCN
ECM	LUM
ECM	BGN
ECM	VCAN
ECM	MMP2
ECM	MMP9
ECM	TIMP1
ECM	TGFBI
ECM	POSTN
CAF	COL1A1
CAF	COL1A2
CAF	COL3A1

CAF	COL5A1
CAF	COL5A2
CAF	FN1
CAF	FAP
CAF	PDGFRB
CAF	ACTA2
CAF	TAGLN
CAF	SPARC
CAF	LUM
CAF	DCN
CAF	THY1
CAF	COL6A1
CAF	COL6A2
CAF	COL6A3
CAF	POSTN

These predefined gene sets were used to compute mean z-score-based ECM and CAF signature scores.

Table 11. Selected untreated PDAC specimens included in the GSE202051 single-nucleus RNA sequencing reanalysis.

Selected untreated PDAC specimens
003
004
007
010T
011
2276
2364
2376
2443
2490
2498
2523
2591
2603
2626
2664
MGHR16
MGHR17

Only treatment-naïve PDAC specimens included in the reanalysis are listed. Specimen selection was based on the processed .h5ad object obtained from GEO.

Table 12. Major-category cell composition and COL5A2 contribution in untreated PDAC specimens from the GSE202051 single-nucleus RNA sequencing cohort.

Major category	Mean proportion	Median proportion	Mean cell count	COL5A2 count sum	Total COL5A2 count	Fraction of total COL5A2 signal
CAF	0.169	0.1605	1017.5556	67162	123748	0.5427
Immune	0.0726	0.0503	460.5556	83	123748	6.71×10^{-4}
Other	0.2072	0.1691	1248.5556	6399	123748	0.0517
Tumor	0.4946	0.5593	2944.3889	4204	123748	0.034
myCAF	0.0566	0.0063	382.5	45900	123748	0.3709

Cell counts and proportions were summarized across major annotated cellular categories in the treatment-naïve PDAC subset. COL5A2 contribution values represent the proportion of total COL5A2-associated signal attributable to each major cellular category, as defined in the reanalysis workflow.
